# Genetic basis of maize kernel oil-related traits revealed by high-density SNP markers in a recombinant inbred line population

**DOI:** 10.1186/s12870-021-03089-0

**Published:** 2021-07-21

**Authors:** Hui Fang, Xiuyi Fu, Hanqiu Ge, Aixia Zhang, Tingyu Shan, Yuandong Wang, Ping Li, Baohua Wang

**Affiliations:** 1 Ministry of Agricultural Scientific Observing and Experimental Station of Maize in Plain Area of Southern Region, School of Life Sciences, Nantong University, Nantong, 226019 People’s Republic of China; 2 Beijing Key Laboratory of Maize DNA Fingerprinting and Molecular Breeding, Maize Research Center, Beijing Academy of Agriculture and Forestry Sciences (BAAFS), Shuguang Garden Middle Road No. 9, Beijing, 100097 China; 3 Nantong Bear Seeds Company, Nantong, 226009 People’s Republic of China

**Keywords:** Maize, Oil-related traits, QTL mapping, Gene-based association analysis

## Abstract

**Background:**

Maize (*Zea mays ssp. mays*) is the most abundantly cultivated and highly valued food commodity in the world. Oil from maize kernels is highly nutritious and important for the diet and health of humans, and it can be used as a source of bioenergy. A better understanding of genetic basis for maize kernel oil can help improve the oil content and quality when applied in breeding.

**Results:**

In this study, a KUI3/SC55 recombinant inbred line (RIL) population, consisting of 180 individuals was constructed from a cross between inbred lines KUI3 and SC55. We phenotyped 19 oil-related traits and subsequently dissected the genetic architecture of oil-related traits in maize kernels based on a high-density genetic map. In total, 62 quantitative trait loci (QTLs), with 2 to 5 QTLs per trait, were detected in the KUI3/SC55 RIL population. Each QTL accounted for 6.7% (*qSTOL1*) to 31.02% (*qBELI6*) of phenotypic variation and the total phenotypic variation explained (PVE) of all detected QTLs for each trait ranged from 12.5% (OIL) to 52.5% (C16:0/C16:1). Of all these identified QTLs, only 5 were major QTLs located in three genomic regions on chromosome 6 and 9. In addition, two pairs of epistatic QTLs with additive effects were detected and they explained 3.3 and 2.4% of the phenotypic variation, respectively. Colocalization with a previous GWAS on oil-related traits, identified 19 genes. Of these genes, two important candidate genes, *GRMZM2G101515* and *GRMZM2G022558*, were further verified to be associated with C20:0/C22:0 and C18:0/C20:0, respectively, according to a gene-based association analysis. The first gene encodes a kinase-related protein with unknown function, while the second gene encodes fatty acid elongase 2 (*fae2*) and directly participates in the biosynthesis of very long chain fatty acids in *Arabidopsis*.

**Conclusions:**

Our results provide insights on the genetic basis of oil-related traits and a theoretical basis for improving maize quality by marker-assisted selection.

**Supplementary Information:**

The online version contains supplementary material available at 10.1186/s12870-021-03089-0.

## Background

Maize (*Zea mays ssp. mays*) is one of the most commonly cultivated cereal crops in the world, and a major source of human food, animal feed and bioenergy. Maize has been used as a model system for plant genetics and solved a lot of uncertain plant biological problems [1]. Maize kernels are composed of approximately 4% fat, 10% protein, and 72% starch and, supplies an energy density of 365 Kcal/100 g [2]. Oil is one of the three main components in maize kernels, whose energy is 2.25 times that of starch [3]. As a mixture, maize oil contains five fatty acids that account for more than 98% of the oil concentration including palmitic (C16:0), stearic (C18:0), oleic (C18:1), linoleic (C18:2) and linolenic (C18:3) acids [4]. Three kinds of unsaturated fatty acids account for 27.5, 51.5 and 1.4%, respectively. The high energy and polyunsaturated fatty acids in maize oil make it a highly-quality edible oil that is healthy for humans. Maize can also be used as biomass energy, which can bring considerable income to industrial production. Therefore, with the increase in oil content in maize kernels, the additional value of maize varieties will certainly increase.

Long-term artificial selection of high-oil maize populations has led to the creation of a series of genetic resources, including Illinois high-oil (IHO) population and Beijing high-oil population [1, 5], which were subsequently widely applied to dissect the genetic architecture of oil biosynthesis in maize kernels. In view of the limitations of analytical methods and molecular markers, the accuracy of QTL mapping based on the biparental population is low, and it is difficult to clone genes decades ago. Subsequently, with the development of sequencing technology [6–8], an increasing number of molecular markers have been applied to QTL mapping, which greatly improves the accuracy of QTL mapping. A large number of chromosomal regions and QTLs affecting oil concentration and fatty acid composition were identified using segregating populations in maize [9–16], and these studies indicated that the oil concentration and fatty acid composition were controlled by a few major genes and many minor genes with mainly additive effects. In addition, epistatic interactions also contribute to variations in oil content in specific populations [15, 16]. Similar results were obtained in two publicly available maize genetic resources, NAM (the nested association mapping population) [17] and AMP508 (association mapping population) [3] based on high-resolution and high-power QTL analysis.

The biological processes of oil synthesis and accumulation are complex in plant seeds and are well known in *Arabidopsis*, in which 120 enzymes and more than 600 genes are involved [18]. However, little is known about maize, and only a few genes related to oil content and fatty acid composition have been cloned [19–22]. For example, the *DGAT1–2* gene, which was cloned by map-based cloning, encodes an acyl-CoA: diacylglycerol acyltransferase, and catalyzed the final step of oil synthesis, which can affect oil and oleic-acid contents [20]. Afterwards, a *DGAT*-based association analysis was carried out to identify the functional loci and develop two PCR-based functional markers [23]. Stearoyl-ACP desaturase (*SAD*) plays a key role in fatty acid biosynthesis, and has been identified in maize by gene-based association analysis [24]. These results suggested that gene-based association mapping was a suitable strategy for revealing the candidate genes underlying QTLs and shortening the time of gene cloning.

In the present study, a RIL population derived from two maize inbred lines, KUI3 and SC55, consisting of 180 individuals was used to: (1) dissect the genetic architecture of oil concentration and fatty acid composition, (2) estimate the number and effects of QTLs and epistatic interactions underlying oil-related traits, and (3) identify candidate genes that control oil related traits.

## Methods

### Genetic materials and field experiments

A RIL population consisting of 180 individuals was developed by a cross between two maize inbred lines KUI3 and SC55. The two parents originated from an previous reported association panel that contained 508 genetically diverse maize inbred lines (AM508) [25]. Hybrid F_1_ was self-pollinated 6 times to produce the F_7_ generation by single-seed descent. All lines and parents were planted in a randomized complete block with two replicates at Beijing and Hainan in 2013. Each family line was grown in a single-row plot (2.5-m rows, 0.67 m between rows), and the planting density was 45,000 plants/ha.

### Measurement of fatty acids in maize kernels

Mature ears were harvested and shelled manually. Fifty kernels were randomly selected, dried for 60 h at 45 °C, ground into powder, and then stored in a desiccator for fatty acid measurement. Lipid was extracted as described by Fang et al. [16]. A HP7890A gas chromatogram (GC) (Agilent Technologies, USA) was employed to analyze the fatty acids compositions. An HP-INNOWAX polyethylene glycol capillary column (30 m × 320 μm × 0.5 μm, Agilent Technologies) was used to separate the samples at 250 °C. The GC was operated at a constant flow pressure of 140.9 kPa, the initial oven temperature of 220 **°**C, with a 16 min isothermal, and then the oven temperatures were increased by 20 **°**C / min to 240 **°**C, with a 5 min isothermal. The FID temperature was 250 **°**C, and the split ratio of nitrogen was 10:1.

Nine distinct fatty acids were measured, including palmitic (C16:0), palmitoleic (C16:1), stearic (C18:0), oleic (C18:1), linoleic (C18:2), linolenic (C18:3), arachidic (C20:0), behenic (C22:0), and lignoceric (C24:0) acids, and the oil content was calculated as the sum of the oils. Another 9 ratio traits were derived from 9 fatty acids: C16:0/C16:1, C16:1/C18:0, C18:0/C18:1, C18:1/C18:2, C18:2/C18:3, C18:0/C20:0, C20:0/C22:0, C22:0/C24:0, SFA/USFA (saturated fatty acid = C16:0 + C18:0 + C20:0 + C22:0 + C24:0; unsaturated fatty acid = C16:1 + C18:1 + C18:2 + C18:3). The detailed protocol was described in Fang et al. [16].

### Analysis of phenotypic data

Phenotypic data processing was performed with R Version 3.6.1 (www.R-project.org). Analysis of variance was performed with the “aov” function in R to evaluate the genotype, environment and replication effect. The model for ANOVA was y = μ + α_g_ + β_a_ + (αβ)_ge_ + γ_ar_ + ε_gar_, where μ represents the grand mean (the total of all the data values from two environments divided by the total sample size), α_g_ represents the effect of the gth line, β_a_ represents the effect of the area, (αβ)_ge_ represents the effect of the line × area interaction, γ_ar_ represents the effect of the area × replicate interaction, and ε_gar_ represents the residual. The broad-sense heritability of each trait was calculated as *H*^2^ = α_g_^2^/(α_g_^2^ + α_ge_^2^/r + α_δ_^2^/ar) [15, 16], where e is equal to 2 environments, r is equal to 2 replications in each environment, α_g_^2^ is the genetic variance, α_ge_^2^ is the interaction of genotype with environment, and α_δ_^2^ is the residual error. The 90% confidence intervals of *H*^2^ were computed.

The function “lmer” in the lme4 package of R was used to fit to a linear mixed model to obtain the best linear unbiased prediction (BLUP) values for each trait of each individual line: y_ijk_ = μ + g_i_ + e_j_ + ε_ijk_, where y_ijk_ is the *k*th phenotypic value of individual *i* in *j*th environment, μ is grand mean of all environments, g_i_ is the *i*th genetic effect, e_j_ is the effect of different environments and ε_ijk_ is the random error. μ was considered a fixed effect and g_i_ and e_j_ were considered random effects [26]. The BLUP values for each line were used for the phenotype description statistics, Pearson correlation coefficient analysis and QTL mapping.

### Construction of genetic map

All family lines, together with their parents, were genotyped with the Illumina MaizeSNP50 BeadChip (Illumina Inc., San Diego, CA, USA), which contains 56,110 single nucleotide polymorphisms (SNPs) covering 19,540 maize genes [27]. The in-house Perl scripts were used to compare the genotypes between parents and the RILs. Missing data, heterozygosity and minor allele frequency for all SNPs and the missing data and heterozygosity for each line were calculated. After quality control, 180 inberd lines with missing data of < 15.0% and heterozygosity of < 8.0% were used for further analysis [28]. A total of 11,841 SNPs were polymorphic with 2372 genetic blocks captured. A modified physical order method as described in Pan et al. [28], was used to construct the genetic linkage map, with all lengths of 1974.8 cM (Fig. S1).

### QTL mapping

Windows QTL Cartographer 2.5 was used to perform QTL mapping for all traits using composite interval mapping [29]. Model 6 of the Zmapqtl module was used to detect QTLs in the whole genome. The scanning interval between markers was set at 2.0 cM, with a 10 cM window size. Forward-backward stepwise regression with five controlling markers was used to control for background from flanking makers. After 1000 permutations, the threshold logarithm of odds (LOD) value to declare putative QTLs was determined at a significance level of *P* < 0.05. The confidence interval of QTL position was estimated with the one-LOD support interval method [30]. The R function ‘lm’ was performed to determine total phenotypic variation explained (PVE) by significant QTLs [4, 16].

### Epistasis analysis

As described as shown in Wen et al. [16, 31], a two-way ANOVA was carried out to estimate the pairwise additive × additive epistatic interactions for all identified QTLs for each trait at *P* < 0.05. The proportion of variance explained by epistasis was evaluated by comparing the residual of the full model that contained all single-locus effects and two-locus interaction effects with that of the reduced model that excluded two-locus interaction effects. In addition, the peak bin markers were used in the epistatic interaction analysis and all the heterozygous genotypes were assigned as missing values for simplicity.

### Gene-based association analysis

The SNPs located in the gene body and regions within 5 kb upstream and downstream of the coding region were extracted from the 1.25 million SNPs with MAF ≥ 0.05 in a panel of 508 maize lines [32]. The associations between all SNPs and oil-related traits were analyzed using a mixed linear model [33] considering the population structure [34] and relative kinship [35]. Bonferroni adjusted significance thresholds (*P* ≤ 0.01/n) of, *P* ≤ 1.6 × 10^− 4^ for *GRMZM2G101515* and *P* ≤ 1.5 × 10^− 4^ for *GRMZM2G022558*, were used to identify significant associations. Linkage disequilibrium (LD) between two sites was calculated with TASSEL 5.0 [35].

### Correlation between gene expression and traits

The analysis of the correlation between gene expression in developing kernels at 15 days after pollination (DAP) [36] and oil-related traits in mature kernels [4] was performed using the R function ‘cor.test’.

### Annotation and gene expression analysis of 19 colocalized genes

Based on the information available in the MaizeGDB (https://www.maizegdb.org), the function of each gene was inferred from orthologues in Arabidopsis or rice. The data of gene expression in developing embryo, endosperm and seeds was obtained in Chen et al. [37].

## Results

### Phenotypic variation, correlation and heritability

Phenotypic variations of the 19 target traits for parents and RILs are shown in Table 1. The mean value of SC55 (5.27%) was higher than that of KUI3 (4.13%) for oil content. The KUI3/SC55 RIL population harbored abundant diversity for most of the investigated phenotypic traits, for which continuous and approximately normal distributions were observed (Fig. S2). The mean of the KUI3/SC55 RIL population based on the BLUP values was close to the mid-parent value for almost all measured traits with transgressive segregation (Table 1), suggesting that both parents harbored the alleles responsible for increasing the oil-related traits. The coefficient of variation (CV) values ranged from 7.39% (C16:0) to 52.10% (C18:0/C18:1), with an average of 33.04% (Table 1). Highly significant effects of genotype, environment and genotype × environment interactions were observed by the ANOVA analysis of all traits except C16:1 (Table 1), indicating that oil-related traits are sensitive to genotypes and environments. Pairwise Pearson’s correlation coefficients of 19 traits revealed that most of the traits showed a significant correlation with each other, with coefficients from 0.17 between C16:0/C18:0 and C18:0/C18:1 to 0.984 between C18:1 and C18:1/C18:2 in the KUI3/SC55 RIL population (Fig. S3). Broad-sense heritability (*H*^2^) was high for all traits, ranging from 0.56 to 0.89, indicating that most of the phenotypic variations were genetically controlled (Table 1).

**Table 1:** Descriptive statistics, broad-sense heritability and ANOVA for 19 oil-related traits in KSC population

Traits	Parents		RILs					ANOVA			
	KUI3^a^	SC55^a^	Mean ± sd	Range	CV (%)^b^	*H*^2 c^	CI (90%) ^d^	Environment	Genotype	Replicate×Environment	Genotype×Environment
OIL	4.13	5.27	4.36 ± 0.50	3.30–5.96	40.62	0.78	0.66–0.85	21.07**	31.51**	0.01	7.07**
C16:0	16.39	16.73	16.63 ± 0.35	15.86–17.61	7.39	0.57	0.37–0.7	1333.86**	85.72**	1.04	36.92**
C16:1	0.17	0.15	0.13 ± 0.01	0.10–0.18	40.41	0.88	0.82–0.91	6.69	2.31**	1.34	0.29
C18:0	2.38	4.00	2.95 ± 0.37	1.94–3.97	48.58	0.76	0.65–0.83	5555.46**	728.56**	1.81	174.13**
C18:1	33.58	31.80	31.02 ± 2.08	25.31–36.89	26.33	0.89	0.84–0.92	61.58**	962.36**	0.70	103**
C18:2	45.54	45.03	46.96 ± 1.85	41.77–51.47	14.71	0.86	0.8–0.9	1744.69**	447.77**	0.83	60.71**
C18:3	1.22	1.22	1.23 ± 0.11	0.96–1.49	30.59	0.83	0.75–0.88	397.62**	253.81**	0.72	42.4**
C20:0	0.61	0.57	0.62 ± 0.04	0.54–0.76	23.93	0.64	0.47–0.75	16,840.67**	239.52**	1.95	86.23**
C22:0	0.19	0.13	0.19 ± 0.02	0.14–0.25	39.89	0.71	0.57–0.8	395.77**	69.89**	0.00	20.22**
C24:0	0.26	0.25	0.26 ± 0.02	0.22–0.33	28.28	0.57	0.37–0.7	73.47**	18.44**	1.37	7.93**
C16:0/C16:1	111.87	117.98	128.18 ± 9.5	101.11–154.04	29.34	0.59	0.39–0.71	420.24**	12.5**	0.13	5.17**
C16:0/C18:0	7.24	4.33	5.82 ± 0.73	4.25–7.79	41.58	0.74	0.61–0.81	1652.16**	677.03**	1.17	178.28**
C18:0/C18:1	0.07	0.13	0.1 ± 0.01	0.06–0.13	52.10	0.74	0.62–0.82	5520**	909.22**	2.25	234.39**
C18:1/C18:2	0.73	0.71	0.66 ± 0.07	0.50–0.90	40.41	0.87	0.81–0.91	907.17**	709.63**	0.95	89.17**
C18:2/C18:3	37.60	37.08	38.71 ± 3.48	31.63–49.76	31.50	0.86	0.78–0.9	1.14	219.1**	1.37	31.12**
C18:0/C20:0	3.76	7.27	4.8 ± 0.69	3.34–7.17	51.54	0.82	0.73–0.87	3172.24**	517.75**	0.78	94.18**
C20:0/C22:0	3.17	4.70	3.43 ± 0.32	2.75–4.30	31.09	0.70	0.56–0.79	225.22**	29.28**	0.05	8.82**
C22:0/C24:0	0.74	0.51	0.71 ± 0.09	0.52–0.91	38.57	0.75	0.63–0.83	14.66	71.37**	0.04	17.67**
SFA/USFA	0.25	0.27	0.26 ± 0.01	0.24–0.28	10.88	0.56	0.35–0.69	2882.21**	94.15**	1.46	41.58**

^a^BLUP values of parents

^b^Coefficient of variation

^c^Broad-sense heritability

^d^90% confidence interval of broad-sense heritability

^⁎^Significant at *P* < 0.05

^⁎⁎^Significant at *P* < 0.01

### Genetic architecture of the oil-related traits

**Fig. 1:**
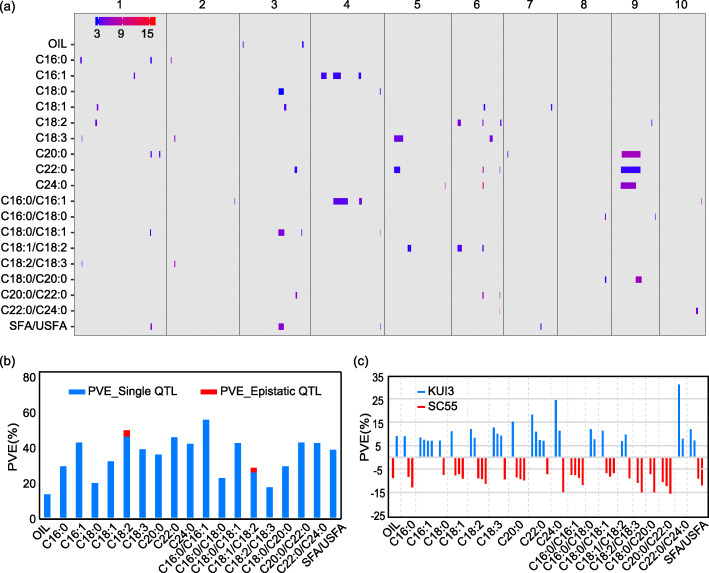
The distribution and effects of single QTL identified for 19 oil related traits in KUI3/SC55 RIL population. **a** Distribution of single QTL on chromosomes. QTL regions across the maize genome are represented by confidence intervals, and LOD values are scaled by color. **b** Total PVE of single (blue bars) and epistatic (red bars) QTLs for each trait. **c** Effect size (represented by PVE) and the origin of the increasing alleles of the identified QTLs. Blue and red bars indicate that increasing alleles come from KUI3 and SC55, respectively

Based on a linkage map of 1974.8 cM, QTLs for 19 oil-related traits were detected in the KUI3/SC55 RIL population. After 1000 permutations, the empirical threshold logarithm of odds (LOD) value for all traits (*P* < 0.05) was 3.2, and the values ranged from 3.2 to 3.6. In total, 62 single QTLs distributed in 38 genomic regions across all chromosomes were detected, with the QTL number per trait ranging from 2 to 5 in the KUI3/SC55 RIL population (Fig. 1a; Table S1). The 1-LOD QTL interval averaged for 9.9 Mb (5.9 cM), with a range from 0.2 to 68.9 Mb (1.2 to 13.8 cM). The phenotypic variation that could be explained by each QTL (PVE) ranged from 6.68% (*qSTOL1*) to 31.02% (*qBELI6*), with an average of 10.3% and the total PVE of all detected QTLs for each trait ranged from 12.5% (OIL) to 52.5% (C16:0/C16:1) (Fig. 1b). Of all these identified QTLs, only 5 had a large effect, with PVE ≥ 15% in three genomic regions on chromosome 6 and 9. The QTL with the largest effect, *qBELI6*, was C22:0/C24:0 on chromosome 6, which was flanked by markers SYN12691 and SYN24474, and accounted for 31.02% of the phenotypic variation. The QTL- *qLIG6* for C24:0 with the second largest effect was on chromosome 6, and accounted for 24.46% of the phenotypic variation, with alleles from KUI3 being responsible for the increasing effect. Additionally, two parents, KUI3 and SC55, harbored similar numbers of favorable alleles at, 29 and 33, respectively (Fig. 1c), suggesting that many favorable alleles existed in regular maize lines with minor effects.

In addition to single QTLs, two pairs of epistatic QTLs referring to 3 loci were detected for two traits, C18:2 and C18:1/C18:2 (Table S2). The two epistatic QTL pairs explained 3.3 and 2.4% of the phenotypic variation. Considering that the number and effect of epistatic QTLs were small, epistatic interactions between two QTLs with additive effects contributed less than additive effects to the genetic basis of oil-related traits in the KUI3/SC55 RIL population.

### QTL clusters

Fourteen QTL clusters were observed in this study, of which 6 covered no less than 3 single QTLs (Fig. 1a; Table S1), and the others covered 2 single QTLs. Specifically, *L25* contained 5 QTLs for 5 oil-related traits on chromosome 6: C18:2, C22:0, C24:0, C18:1/C18:2 and C20:0/C22:0. The PVE of these QTLs ranged from 8.9 to 24.46%, of which two were main-effect QTLs (Fig. S4a). On chromosome 1 and 9, there were two loci harboring 4 QTLs: *L6* for C16:0, C20:0, C18:0/C18:1, and SFA/USFA and *L34* for C24:0, C22:0, C20:0, and C18:0/C20:0, respectively. All these QTLs were minor-effect QTLs except *qARA9*, whose PVE ranged from 6.68 to 15.22%. The region of *L34* spanned 25.8–100.4 Mb and was much larger than that of *L6* (257.6–264.5 Mb), as a result of low-frequency recombination events occurring in the interval of *L34*. The other 3 loci, i.e., *L12*, *L20* and *L28*, contained 3 QTLs and were located on chromosome 3, 4 and 6, respectively. *L28* spanned a small region from 164.0 to 165.4 Mb, contained *qBELI6*, *qBEH6–2* and *qARBE6–2* and could explain 31.02, 10.82 and 15.36% of the phenotypic variation, respectively, which makes it a valuable target for further gene cloning. The spanning interval of *L20* was 1.5 Mb (237.5–239.0 Mb), which was much smaller than that of *L12*, whose interval was more than 20 Mb. All the 6 QTLs for *L12* and *L20* had minor effects, with PVEs ranging from 6.75 to 11.91%.

Interestingly, QTL-gene colocalization identified 2 known genes falling within 2 loci for *L25* and *L19* (Fig. S4). The *DGAT1–2* gene [20], which encodes an acyl-CoA: diacylglycerol acyltransferase and catalyzes the final step of oil synthesis, is located in the interval of *L25*, and might be the candidate gene (Fig. 4a). Additionally, the *FAD2* gene encoding fatty acid dehydrogenase colocalized with *L19*, which covered *qPAE4–3* and *qPALE4–2* (responsible for C16:1 and C16:0/C16:1, respectively) (Fig. S4b).

### Mining of candidate genes for oil-related traits by linkage and gene-based association analysis

**Fig. 2:**
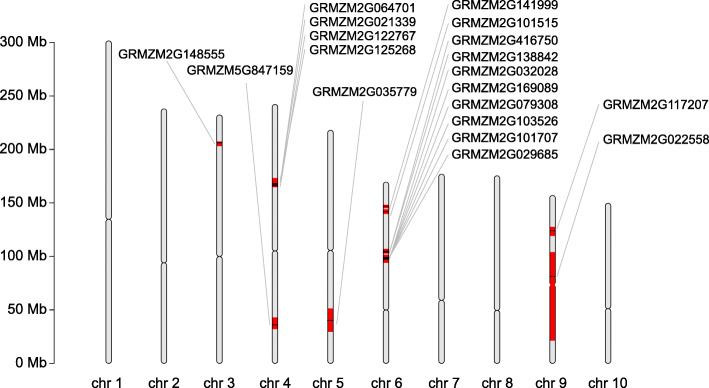
Co-localization of QTLs and known genes for oil-related traits. All 10 maize chromosomes (Chr 1–10) were depicted to scale (Mb, million base pairs). Red region indicated the confidence interval region of QTLs and the black lines indicated the position of genes

Combined with a previous report about a GWAS for 21 oil-related traits [4], 19 (25.7%) of the 74 candidate genes were detected in this study based on physical position (Fig. 2; Table S1). These genes covered 10 loci that have the potential to affect oil biosynthesis and accumulation in maize kernels. Of the 19 genes, 4 encoded enzymes involved lipid metabolism reactions that directly regulated the lipid synthesis and metabolism including fatty acid desaturase 2 (*FAD2*), fatty acid elongase 2 (*FAE2*), diacylglycerol acyltransferase (*DGAT1–2*) and Myristoyl-acyl carrier protein thioesterase (Fig. S5; Table S4). four genes were annotated as enzymes involved in other metabolism reactions, such as aldehyde dehydrogenase, Ser/Thr protein phosphatase, acid phosphatase and alpha/beta-Hydrolases. One gene was annotated as transcription factor. The proteins encoded by remaining 10 genes were classified as chaperonin protein, ribosomal protein, zinc finger, G protein, cytochrome P450 and proteins with unknow function (Fig. S5). In addition, combined with the published RNA-seq data, we found that 94.7% (18/19) of these genes expressed in developing embryo, endosperm and seeds except *GRMZM2G141999* (Fig. S6; Table S4). Eight genes were highly expressed in developing embryo at various stages, which indicated the potential roles in lipid synthesis and metabolism, because the embryo was the main site of oil accumulation.

**Fig. 3:**
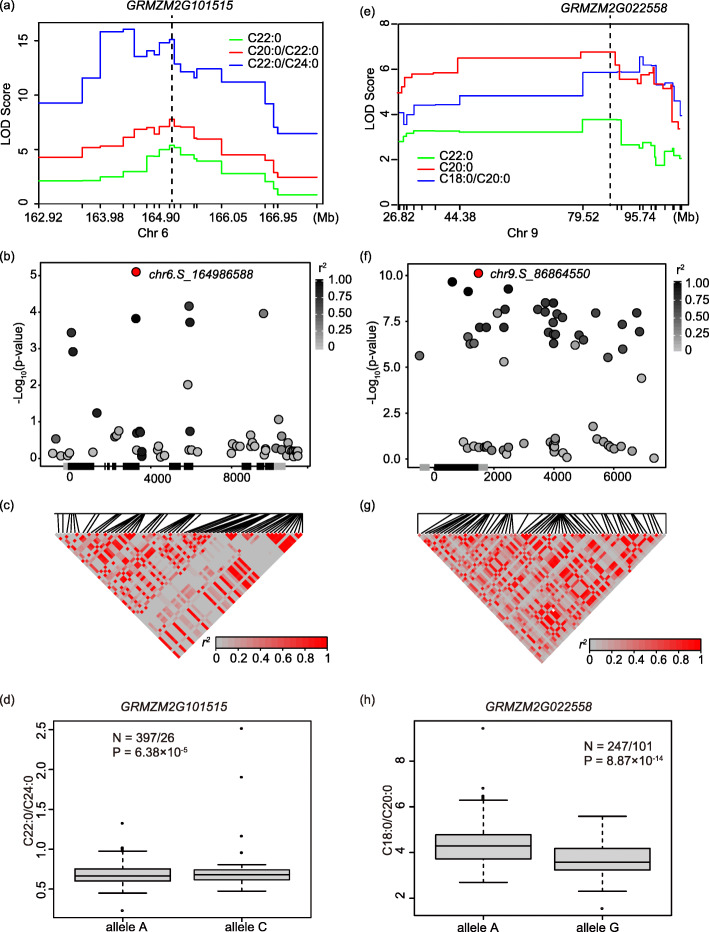
Candidate genes for *L28* and *L34*. **a** LOD profiles of the identified QTL bins for *L28* and *GRMZM2G101515* were colocalized with a QTL cluster. The dashed lines show the physical positions of genes. **b** Candidate-gene association analysis for *GRMZM2G101515*. The most significant locus is shown in red. The intensity of gray shading indicates the extent of LD (*r*^2^) between the most significant locus and the other variants identified in this region. The gene struct.is shown on the x-axis. The black and light-gray shading represents exons and UTRs, respectively. **c** The linkage disequilibrium (LD) patterns of all identified variants (MAF ≥ 0.05) in genes *GRMZM2G101515*. **d** The effect of peak locus for *GRMZM2G101515* in an association panel. **e** LOD profiles of the identified QTL bins for *L34* and *GRMZM2G022558* were colocalized with a QTL cluster. **f** Candidate-gene association analysis for *GRMZM2G022558*. **g** The linkage disequilibrium (LD) patterns of all identified variants (MAF ≥ 0.05) in genes *GRMZM2G022558*. **h** The effect of peak locus for *GRMZM2G022558* in an association panel

**Fig. 4:**
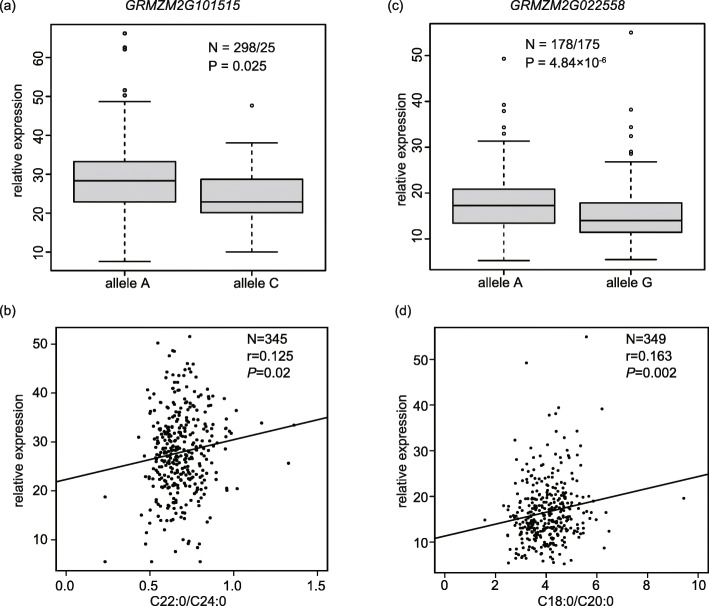
*GRMZM2G101515* and *GRMZM2G022558* alter the ratio of fatty acids in maize kernel. **a** Comparison of the relative expression at peak locus alleles A and C for *GRMZM2G101515*. **b** Correlation of C22:0/C24:0 with the relative expression of *GRMZM2G10151* in kernels at 15 DAP. **c** Comparison of the relative expression at peak locus allele A and G for *GRMZM2G022558*. **d** Correlation of C18:0/C20:0 with the relative expression of *GRMZM2G022558* in kernels at 15 DAP

Coincidentally, two of the 8 genes highly expressed in embryo were located in two QTL clusters in physical position, *L28* on chromosome 6 and *L34* on chromosome 9, which fell in the peak bin of the most colocalized QTLs (Fig. 3a, e), and were considered important candidate genes. *L28* contained 3 QTLs, namely, *qBELI6* for C22:0/C24:0, with the largest PVE of 31.02%, moderate effect QTL-*qBEH6–2* for C22:0 and major QTL-*qARBE6–2* for C20:0/C22:0, encompassing the *GRMZM2G101515* gene, which can encode a protein with an unknow function (Fig. 3a). To further explore the association between the gene and oil-related traits, 62 SNPs were extracted in the gene body and region within 5 kb upstream and downstream of the *GRMZM2G101515* gene from 1.25 million high-quality SNPs with MAF ≥ 0.05 in 508 maize inbred lines [32]. A marker-trait association analysis with these SNPs using a mixed linear model identified 4 significant loci associated with C22:0/C24:0 at *P* ≤ 1.6 × 10^− 4^ (Fig. 3b, Table S3). The most significant SNP, *chr6.S_164986588* for alleles A and C, was located on exon 5 at *P* = 7.91 × 10^− 6^, which can give rise to a change in amino acids for glutamate (T) to threonine (P). The LD between *chr6.S_164986588* and the other three SNPs ranged from 0.34 to 1 (Fig. 3c). The lines with allele C have a higher ratio of C22:0/C24:0 than those with allele A (Fig. 3d). Meanwhile, the lines harboring allele A expressed *GRMZM2G101515* at slightly high levels at *P* = 0.025 (Fig. 4a), and the gene expression and the ratio of C22:0/C24:0 showed a weak correlation (Fig. 4b). These results showed that the expression difference of the *GRMZM2G101515* gene might affect the ratio of C22:0/C24:0.

In addition, 3 of 4 QTLs contained in *L34*, namely, *qBEH9* for C22:0, *qARA9* for C20:0 and *qSTAR9* for C18:0/C20:0, colocalized with the *GRMZM2G022558* gene (Fig. 3e). This gene encodes fatty acid elongase 2 (*fae2*), which is involved in the biosynthesis of very long-chain fatty acids in *Arabidopsis* [38], and is incorporated into a variety of plant lipids. Similarly, from a 1.25 million SNP database of 508 maize inbred lines, we extracted 66 SNPs with MAF ≥ 0.05 spanning from 5-kb up- to downstream of *fae2* coding region, and then 35 SNPs that associated with C18:0/C20:0 were identified by marker-trait association analysis, including 2 in the 5’UTR, 6 in exon1, 1 in the 3′ UTR and the rest in the region behind the 3’UTR. The peak signal, *chr9.S_86864550* in exon 1, whose *P*-value reached 7.52 × 10^− 11^(Fig. 3f, Table S3), was in LD with almost all the other significant SNPs, which can lead to amino acid changes (Fig. 3g). Lines harboring allele A were significantly greater than those harboring G at the ratio of C18:0/C20:0 (Fig. 3h). Meanwhile, the expression of *fae2* in lines with allele A was distinctly higher than that in lines with allele G (Fig. 4c) and the expression level was correlated with the ratio of C18:0/C20:0 at *P* = 0.002 (Fig. 4d). These results indicated that changes in the expression of *fae2* could change phenotypes.

## Discussion

### Genetic components of oil-related traits in maize kernels

Maize oil is a compound made up of different kinds of fatty acids. Previous studies have shown that oil-related traits are quantitative traits controlled by multiple genes [9–17], which was also revealed by the finding that all traits followed normal distributions and showed transgressive segregation in this work. Our study detected 62 QTLs at 38 loci, of which only 5 were major QTLs, with PVE ≥ 15%. Therefore, two contrasting genetic architectures were found for 19 oil-related traits. Three fatty acid traits, C20:0, C22:0, and C24:0 and two ratio traits C20:0/C22:0 and C22:0/C24:0, were controlled by a single major QTL plus some small-effect QTLs, while the others were controlled by many small-effect QTLs. It is worth noting that only two minor-effect QTLs were detected for oil content in the present study, which is in keeping with a recent report [16], while 6–16 QTLs were identified in high-oil populations [11, 13–15], 22 QTLs identified in the NAM population [17] and 26 loci associated with oil content throughout a GWAS analysis [4]. The application of different mapping populations gave rise to differences in QTL numbers and effects. In the present study, subtle variations in oil content between two parents, KUI3 (4.13%) and SC55 (5.27%), were observed. Two QTLs contributed to 12.5% of the phenotypic variation in oil content in the KUI3/SC55 RIL population. The PVE for oil content was more than 50% in high-oil populations [15, 39]. Favorable allele accumulation is a route for increasing oil concentration, and high-oil maize lines have more favorable alleles, some of which have main effect; therefore, an increasing number and effect of QTLs were identified in the biparental population constructed by high-oil lines. Nevertheless, favorable alleles also existed in regular maize lines according to the founding in this study. In addition, varying environments could also influence the number of detected QTLs.

Another remarkable finding in this work is that two pairs of epistatic interaction QTLs with additive effects were identified for two oil-related traits. However, they presented limited contributions to increasing the fatty acids composition. This result was consistent with a few previous reports [15, 16]. As an example, 2–7 pairs of epistatic QTLs were detected for oil content and 5 fatty acid compositions in high-oil maize [15]. The proportion of total phenotypic variance explained by all epistatic QTLs ranged from 5.2 to 12.6% for each trait. Similar results were obtained in rapeseed, rice, peanut and wheat [40–43], demonstrating that epistasis could make a substantial contribution to variation in complex quantitative traits in different crops. The magnitudes of individual QTLs with additive effects and the percentage of total phenotypic variation explained by individual QTLs were greater than those of epistatic QTLs, indicating that additive effects rather than epistatic effects played a crucial role in contributing to the genetic basis of oil-related traits in the KUI3/SC55 RIL population.

### Colocalization of oil-related QTLs identified in this study with previous studies

The mining of oil-related QTLs is beneficial for a better understanding of oil biosynthesis and accumulation in maize kernels. In comparison with previous studies [11, 13–17], 75.8% (47/62) of all identified QTLs in this study were previously reported based on the B73 reference genome version 2, indicating the reliability and accuracy of the results. All 15 newly identified QTLs had moderate effects and were distributed on 8 chromosomes (except for chromosome 5 and 6), and the PVE ranged from 6.75% (*qSTOL4*) to 11.94% (*qPAST8*), which revealed the specificity of the genetic background from the two parents. Considering the physical position of all QTLs, 26.3% (10/38) of loci were freshly verified in the current study, including two QTL clusters, *L20* and *L33*, located on chromosome 4 for C18:0, C18:0/C18:1, and SFA/USFA and chromosome 8 for C16:0/C18:0 and C18:0/C20:0, respectively. Pleiotropy and close linkage could cause trait correlations and lead to colocalization of QTLs, which means that a few QTLs controlling different traits were identified in the same genomic regions [44]. These two new loci supported by multiple QTLs are credible and have the potential to serve breeding. Moreover, the reasons for colocalization of the two loci require further study.

The two candidate genes, *GRMZM2G101515* and *GRMZM2G022558*, were associated with C20:0/C22:0 and C18:0/C20:0, respectively. The former encodes a kinase-related protein with unknow function, and in *Arabidopsis*, it was annotated as an RNA polymerase II degradation factor-like protein. No reports have shown that this gene is related to lipid metabolism. Given that this gene is not in the lipid metabolism pathway, it probably regulates lipid metabolism in an indirect way. In addition, the most significant SNP, *chr6.S_164986588*, is not necessarily the functional site of the gene. In most cases, the change of a single amino acid is not enough to change the protein function. The expression of *GRMZM2G101515* is related to the phenotype, which means that the phenotype is likely to be regulated by gene expression. The real functional site is possible to be some transposons or structural variations undiscovered by the next-generation sequencing technology, which are in LD with the significant SNPs, just like the way of *ZmNAC111* and *ZmVPP1* [45, 46] work. The second encodes fatty acid elongase 2 (fae2), which can elongate fatty acyl-CoAs to produce C20-C24 acyl-CoAs and then further produce long-chain fatty acids, and it directly participates in the biosynthesis of very long-chain fatty acids in *Arabidopsis* [18]. Similarly, although many significant SNPs were identified to be associated with C18:0/C20:0, it’s hard to determine the functional site of *GRMZM2G022558* gene. The significant correlation between the phenotype and gene expression in the association panel indicated the possibility that expression regulated the phenotype.

### QTL application in the improvement of maize oil

QTL mapping is a classical strategy to identify loci for complex quantitative traits of interest. The ultimate goal of QTL mapping is to clone the causal genes for further application in trait improvement. It usually takes a long time to obtain genes underlying QTLs by constructing near-isogenic lines in maize [47–49], rice [50–52], wheat [53, 54] and other plants [55, 56]. Combining linkage analysis and GWAS can greatly shorten the journey [57, 58], and gene-based association studies can help identify the favorable allele. These QTLs or genes have the potential to contribute crop improvement by marker-assisted selection. To date, a large number of QTLs for different traits in multiple species have been detected, and some of which have actually been applied to crop improvement [59–61]. However, little is applied in oil improvement for maize kernels apart from the *DGAT1–2* gene [62]. In detail, Hao et al. [62] transferred the favorable allele of *DGAT1–2* from the high-oil inbred line (By804) into two parents of Zhengdan958 using marker-assisted backcrossing and successfully increased the oil content of the improved Zhengdan958 without a change in grain weight. In the present study, 5 major QTLs were identified, and 3 of these QTLs were isolated by joint gene-based association analysis, and two candidate genes were verified. In addition, these QTLs were mainly additive in the KUI3/SC55 population, which may accelerate molecular breeding by pyramiding the favorable maize alleles of these detected QTLs or by genomic selection.

## Conclusions

In the present study, QTL mapping for 19 oil-related traits was conducted with high-density SNP markers in the KUI3/SC55 RIL population. A large number of QTLs regulating oil content and fatty acid composition were identified, most of which were moderate effect QTLs. Two contrasting genetic architecture were revealed for 19 oil-related traits. Of these traits, only five harbor a major QTL, reflecting the complex nature of oil-related traits. In addition, additive effects rather than epistatic effects played a crucial role in contributing to the genetic basis of oil-related traits in the KUI3/SC55 RIL population. Two genes, *GRMZM2G101515* and *GRMZM2G022558*, were further verified to be associated with C20:0/C22:0 and C18:0/C20:0, respectively, by gene-based association analysis. The first gene encodes a kinase-related protein with unknown function, which is likely to act as a regulator to influence the genes involved in oil biosynthesis and metabolism pathway. While the second gene encodes fatty acid elongase 2 (fae2) and directly participates in the biosynthesis of very long-chain fatty acid in *Arabidopsis*, so that it can regulate the ratio of C18:0/C20:0 by affecting the content of both fatty acids in a direct way. In total, these findings provide insights into the genetic architecture of oil-related traits and an opportunity to increase oil content and improve oil quality in maize kernels.

## Supplementary Information

**Additional file 1: Figure S1.** Recombination bin map of 180 lines in the KUI3/SC55 RIL population. **Figure S2.** Phenotypic distributions of 19 oil-related traits in the KUI3/SC55 RIL population. **Figure S3.** Pearson correlation coefficients (upper right) for the oil-related traits and -log_10_ (*P*-value) of the Pearson correlation (bottom left). **Figure S4.** LOD profiles for the QTL clusters that colocalized with previously cloned genes. **Figure S5.** Functional category annotations for 19 colocalized genes. **Figure S6.** Heat map of gene expression for 19 colocalized genes in developing embryo, endosperm, and seed at various developing stages.

**Additional file 2: Table S1.** Single QTLs for 19 oil-related traits identified in this study.

**Additional file 3: Table S2.** Epistasis interactions between pairs of QTLs with additive effects.

**Additional file 4: Table S3.** Associations between *GRMZM2G101515*, *GRMZM2G022558* polymorphisms and two oil-related traits in 508 maize inbred lines.

**Additional file 5: Table S4.** The functional annotation and the RPKM values of gene expression for the 19 colocalized genes.

## Data Availability

The datasets supporting the conclusions of this article are included within the article and its supplementary files.

## Abbreviations

RIL: Recombinant inbred line
PVE: Phenotypic variation explained
C16:0: Palmitic acids
C16:1: Palmitoleic acids
C18:0: Stearic acids
C18:1: Oleic acids
C18:2: Linoleic acids
C18:3: Linolenic acids
C20:0: Arachidic acids
C22:0: Behenic acids
C24:0: Lignoceric acids
SFA: Saturated fatty acids
USFA: Unsaturated fatty acids
BLUP: Best linear unbiased prediction
QTL: Quantitative trait locus/loci
LOD: logarithm of odds
LD: Linkage disequilibrium
DAP: Days after pollination
CV: Coefficient of variation
MAF: Minor allele frequency
