# Supplementary material for: Genetic basis of maize kernel oil-related traits revealed by high-density SNP markers in a recombinant inbred line population
Source: BMC Plant Biol. 2021 Jul 21;21:344. doi: 10.1186/s12870-021-03089-0 (PMC8293480; doi:10.1186/s12870-021-03089-0)
Supplement: Supplementary file 1 — Additional file 1: Figure S1. Recombination bin map of 180 lines in the KUI3/SC55 RIL population. Figure S2. Phenotypic distributions of 19 oil-related traits in the KUI3/SC55 RIL population. Figure S3. Pearson correlation coefficients (upper right) for the oil-related traits and -log10 (P-value) of the Pearson correlation (bottom left). Figure S4. LOD profiles for the QTL clusters that colocalized with previously cloned genes. Figure S5. Functional category annotations for 19 colocalized genes. Figure S6. Heat map of gene expression for 19 colocalized genes in developing embryo, endosperm, and seed at various developing stages. [file 12870_2021_3089_MOESM1_ESM.docx]

**Genetic basis of maize kernel oil-related traits revealed by high-density SNP markers in a recombinant inbred line population**

**Hui Fang^a,1^, Xiuyi Fu^b,1^, Hanqiu Ge^a^, Aixia Zhang^a^, Tingyu Shan^a^, Yuandong Wang ^b, *^, Ping Li^a, c*^, Baohua Wang^a,*^**

*^a^* *Ministry of Agricultural Scientific Observing and Experimental Station of Maize in Plain Area of Southern Region, School of Life Sciences, Nantong University, Nantong, 226019 P.R. China*

*^b^ Maize Research Center, Beijing Academy of Agriculture & Forestry Sciences (BAAFS), Beijing Key Laboratory of Maize DNA Fingerprinting and Molecular Breeding, Shuguang Garden Middle Road No. 9, Beijing 100097, China*

*^c^Nantong Bear Seeds Company, Nantong, 226009 P.R. China*

*^1^* *These authors contributed equally to this work.*

**^*^** *Corresponding author.*

*E-mail address*

*wyuandong@126.com* (Yuandong Wang); *pingli6@hotmail.com (Ping Li);* *bhwang@ntu.edu.cn (Baohua Wang)*

The following Supporting Information is available for this article:

**Figure S1** Recombination bin map of 180 lines in the KUI3/SC55 RIL population.

**Figure S2** Phenotypic distributions of 19 oil-related traits in the KUI3/SC55 RIL population.

**Figure S3** Pearson correlation coefficients (upper right) for the oil-related traits and -log_10_ (*P*-value) of the Pearson correlation (bottom left).

**Figure S4** LOD profiles for the QTL clusters that colocalized with previously cloned genes.

Figure S5 Functional category annotations for 19 colocalized genes.

Figure S6 Heat map of gene expression for 19 colocalized genes in developing embryo, endosperm, and seed at various developing stages.

**Table S1** Single QTLs for 19 oil-related traits identified in this study.

**Table S2** Epistasis interactions between pairs of QTLs with additive effects

**Table S3** Associations between *GRMZM2G101515*, *GRMZM2G022558* polymorphisms and two oil-related traits in 508 maize inbred lines.

Table S4 The functional annotation and the RPKM values of gene expression for the 19 colocalized genes.


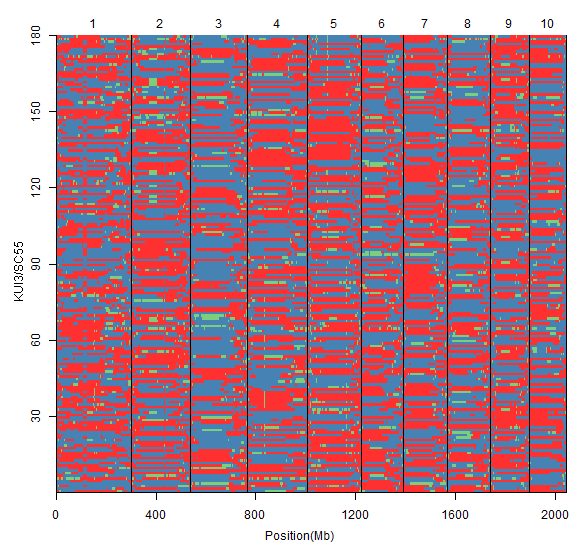


Figure S1 Recombination bin map of 180 lines in the KUI3/SC55 RIL population. The red and blue represent genotypes of KUI3 and SC55, respectively, and green represents heterozygous genotype.


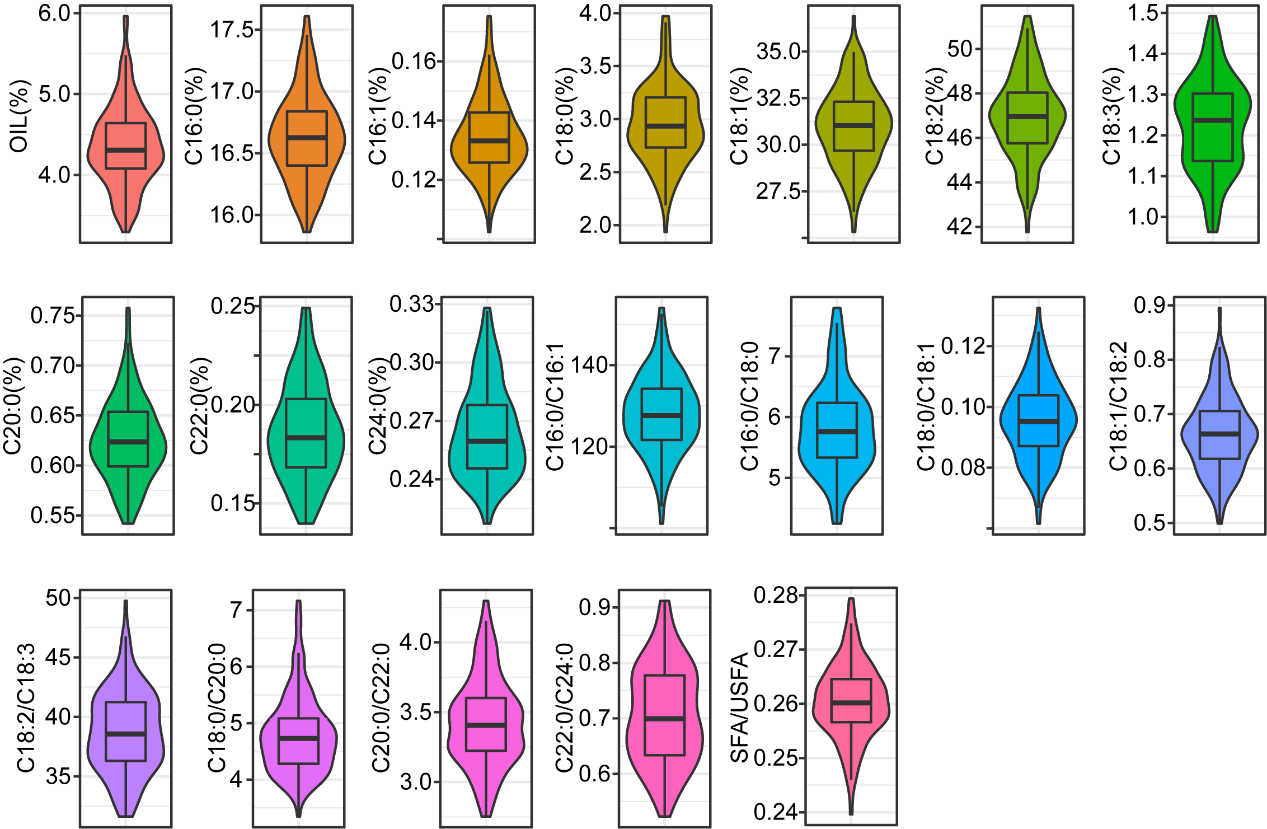


Figure S2 Phenotypic distributions of 19 oil-related traits in the KUI3/SC55 RIL population.


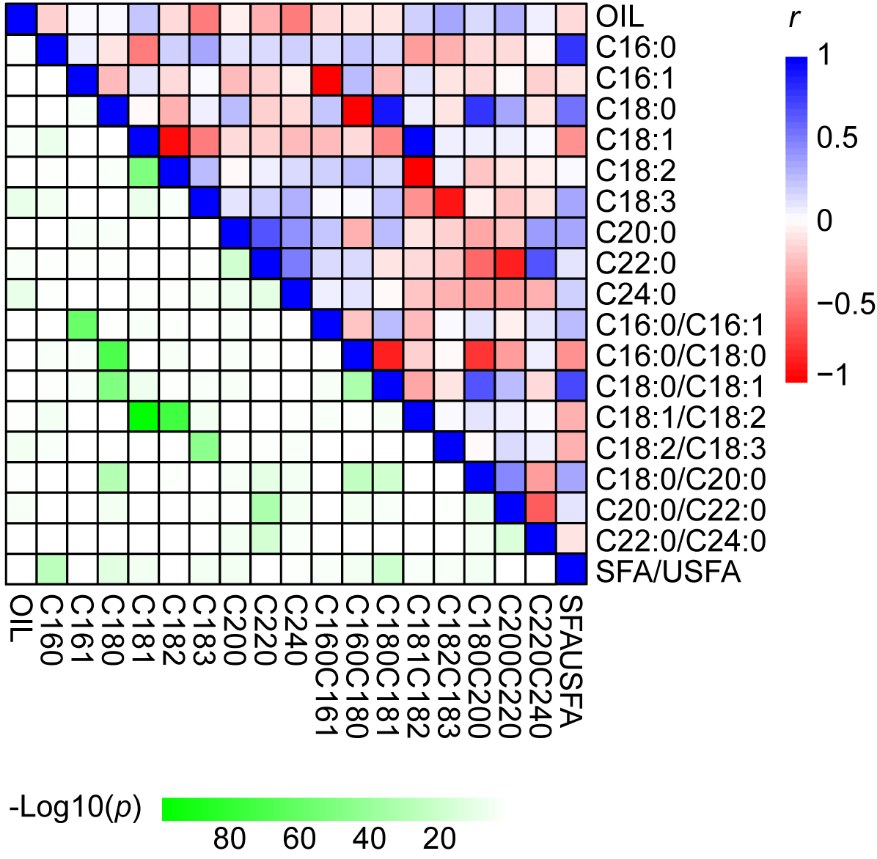


Figure S3 Pearson correlation coefficients (upper right) for the oil-related traits and -log_10_ (*P*-value) of the Pearson correlation (bottom left).


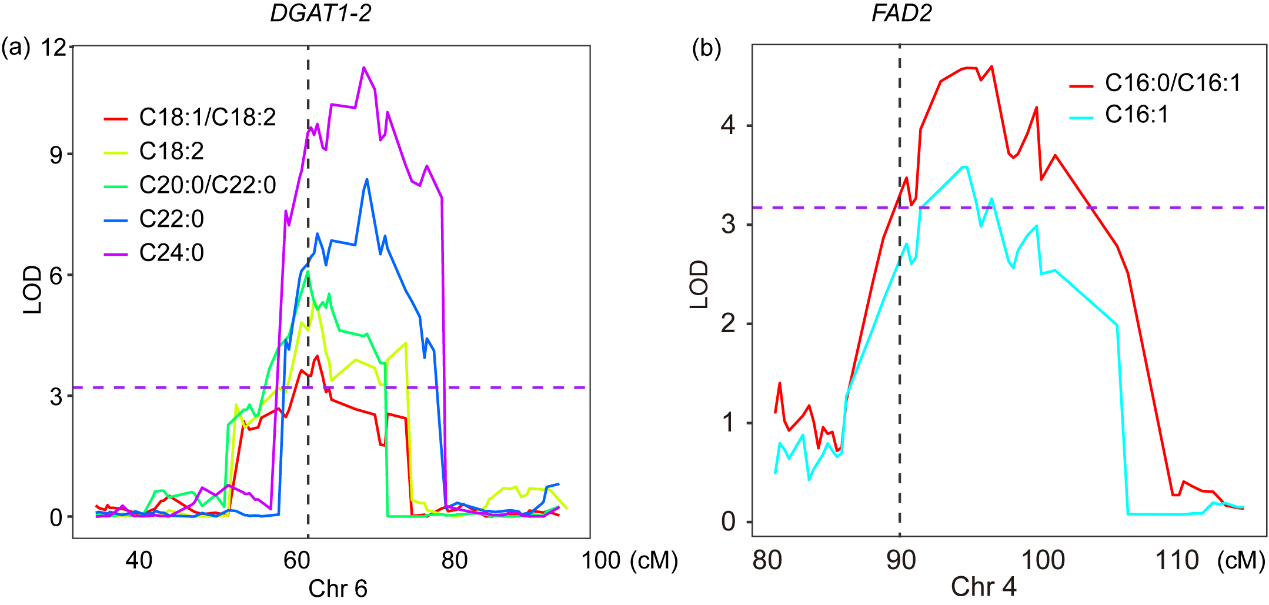


Figure S4 LOD profiles for the QTL clusters that colocalized with previously cloned genes. The vertical dashed lines indicate the position of two genes, *DGAT1-2* (a) and *FAD2* (b).


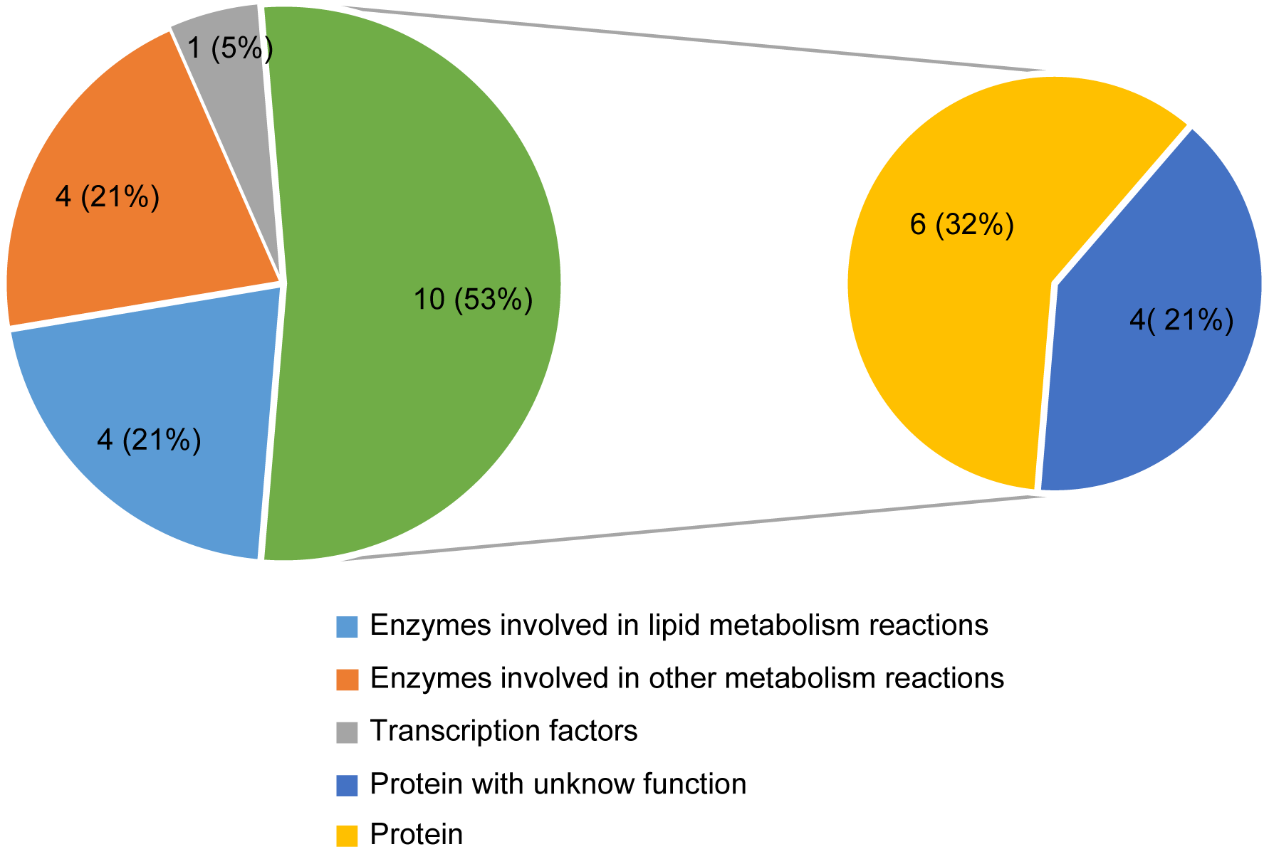


Figure S5 Functional category annotations for 19 colocalized genes.


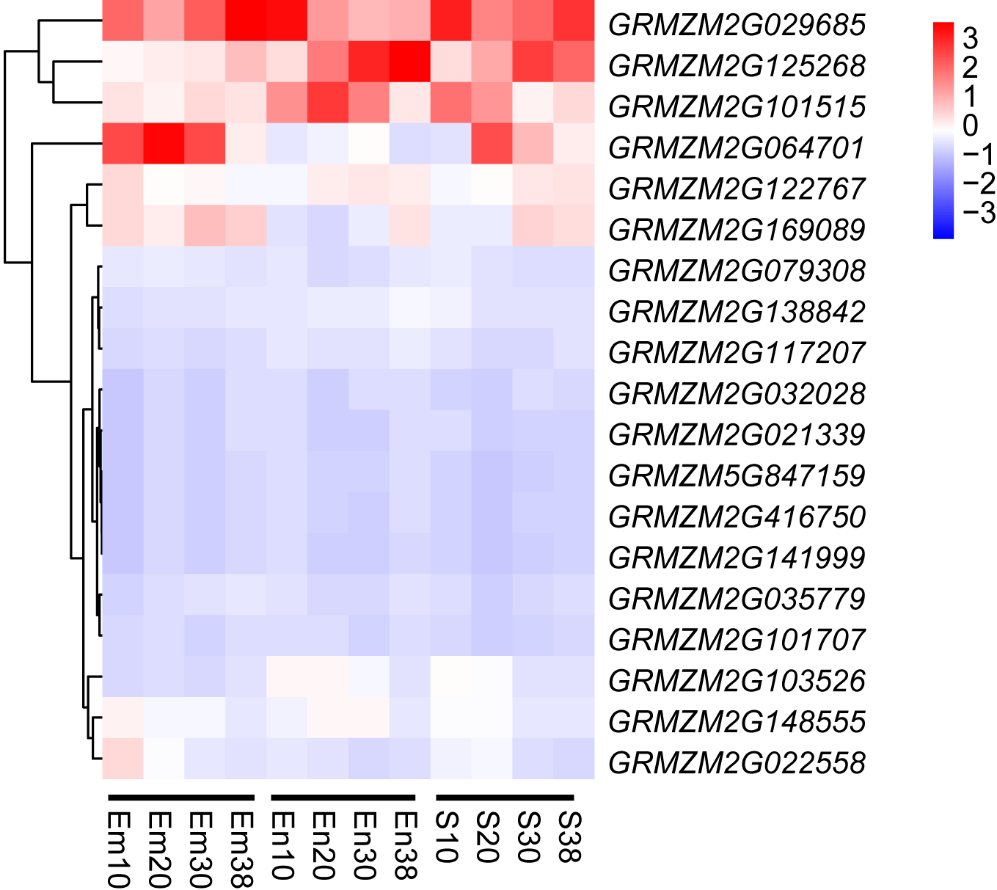


Figure S6 Heat map of gene expression for 19 colocalized genes in developing embryo, endosperm, and seed at various developing stages. For each gene, the RPKM value normalized by the maximum value of all RPKM values of the gene over all time points is shown.
